# Shannon Entropy Used for Feature Extractions of Optical Patterns in the Context of Structural Health Monitoring

**DOI:** 10.3390/e25081207

**Published:** 2023-08-14

**Authors:** Wendy Garcia-González, Wendy Flores-Fuentes, Oleg Sergiyenko, Julio C. Rodríguez-Quiñonez, Jesús E. Miranda-Vega, Daniel Hernández-Balbuena

**Affiliations:** 1 Engineering Faculty, Universidad Autónoma de Baja California, Mexicali 21280, BC, Mexico; wendy.garcia26@uabc.edu.mx (W.G.-G.); flores.wendy@uabc.edu.mx (W.F.-F.); julio.rodriguez81@uabc.edu.mx (J.C.R.-Q.); dhernan@uabc.edu.mx (D.H.-B.); 2 Engineering Institute, Universidad Autónoma de Baja California, Mexicali 21100, BC, Mexico; srgnk@uabc.edu.mx; 3 Department of Computer Systems, Tecnológico Nacional de México, IT de Mexicali, Mexicali 21376, BC, Mexico

**Keywords:** machine learning, data augmentation, sensor data processing, technical vision system, optical patterns, random process, entropy

## Abstract

A novelty signal processing method is proposed for a technical vision system (TVS). During data acquisition of an optoelectrical signal, part of this is random electrical fluctuation of voltages. Information theory (IT) is a well-known field that deals with random processes. A method based on using of the Shannon Entropy for feature extractions of optical patterns is presented. IT is implemented in structural health monitoring (SHM) to augment the accuracy of optoelectronic signal classifiers for a metrology subsystem of the TVS. To enhance the TVS spatial coordinate measurement performance at real operation conditions with electrical and optical noisy environments to estimate structural displacement better and evaluate its health for a better estimation of structural displacement and the evaluation of its health. Five different machine learning (ML) techniques are used in this work to classify optical patterns captured with the TVS. Linear predictive coding (LPC) and Autocorrelation function (ACC) are for extraction of optical patterns. The Shannon entropy segmentation (SH) method extracts relevant information from optical patterns, and the model’s performance can be improved. The results reveal that segmentation with Shannon’s entropy can achieve over 95.33%. Without Shannon’s entropy, the worst accuracy was 33.33%.

## 1. Introduction

Modern society requires infrastructure to perform indispensable activities such as transportation, communication, power grid, and water supply systems. These urban infrastructures (UI) are necessary to sustain a city’s economy. The rapid urban growth allows testing the strengths of the civil infrastructure (CI). Traffic loads and natural hazards are factors that can cause deterioration of the UI. To pursue sustainable development goals, it is necessary to consider the monitoring and control of current infrastructure. Urban sensing deals with collecting relevant information about the urban environment to develop early warning systems to make sustainable urban systems through technology. Different types of data sets of large amounts of information can be gathered, like air quality [1], traffic patterns [2], and CI [3], just to mention a few examples.

The most common variables studied in CI that need adequate maintenance are related to energy, transportation, and building. The datasets taken from CI are urban area, population density, energy utilization by each consumer, etc. All this information is collected and used to understand complex problems that are hard to solve, like the challenge of aging infrastructure. To face this challenge, a TVS is proposed to capture optical signals to create datasets from particular behaviors of the laser beam reflected from a CI.

Optimizing maintenance activities of a key UI is an important task that should be adopted to ensure good performance under challenging conditions. Sustaining key infrastructures requires constant monitoring for the safety of citizens. These elements must follow strict safety requirements to avoid stopping the economy. Adequate maintenance of these infrastructures can save lives. For these reasons, preserving civil and industrial infrastructures through programmed maintenance is important. The field of structural health monitoring (SHM) is a convenient and organized way to address the current challenges. The methodologies addressed by SHM are designed to develop technologies for monitoring and analyzing data to prevent damage to infrastructure. SHM can help us to evaluate the risks and manage assets better.

Nowadays, many engineers have implemented technologies to solve problems related to damage detection. The current technology improved diagnostic accuracy by identifying problems to make decisions objectively. On the other hand, sensor information is one of the most important elements in quantifying risk. This allows for defining strategies to minimize the likelihood of critical damage. To collect the information from structures there are different technologies based on materials such as fiber Bragg grating (FBG) for strain sensor applications in road [4], piezoelectric nanofiber membranes sensor based on PAN/BaTiO_3_ (polyacrylonitrile and flexible barium titanate) [5], Carbon nanotubes (CNTs) [6]. FBG is a technology based on optical fiber that reflects certain wavelengths and transmits others. This material also be used for sensing applications. PAN/BaTiO3 is a novel nanomaterial proposed for electromechanical conversion in SHM tasks due to its strong piezoelectricity capacity. A CNT is a tiny hollow tube made of cylindrical molecules of carbon that is widely used in many fields of science due to its electromechanical and thermal properties. In the field of SHM, the CNT is used for measuring the strain, stress, load, temperature, displacement, and pressure.

Recent literature based on numerical and experimental models to address classification problems can be solved using supervised and unsupervised machine learning techniques. These can be described as follows. The following authors [7] proposed a methodology based on the acceleration and shear time histories evaluated on the rails. The work is treated as a binary classification. The methodology proposed could automatically distinguish a defective wheel from a healthy one. The development of an easy-to-implement, low-cost monitoring system is a relevant contribution. The continuous wavelet transform (CWT) model was used as a feature extractor from acquired responses.

A general ML framework to deal with the railway wheel flats identification can be consulted in [8]. They deal with damage identification based on the acceleration measurements on the rails. A numerical approach was performed to evaluate whether the number of sensors used to detect and classify wheel flats. An autoregressive (AR) model was performed as a feature extractor to take meaningful information from measurements.

The following research [9] studies the different vibration-based damage detection methods, such as fundamental modal examination, local diagnostic method, non-probabilistic methodology, and the time series method.

A Singular spectrum analysis (SSA) is a nonparametric method for analyzing time series. This tool can enhance the sensitivity of the acceleration signals. SSA uses time history data obtained from each sensor separately, and the singular value decomposition (SVD) is performed on the Hankel matrix formed [10]. The work [11] was focused on detecting and identifying damage in a structure in an online framework. They proposed a methodology for real-time based on recursive singular spectrum analysis (RSSA). According to the findings, RSSA facilitates the monitoring of structural systems and real-time data processing through acceleration data using single and multiple sensors. The exact damage instant can be identified by extracting damage-sensitive features from measurements.

The authors [12] give a broader discussion of first-order perturbation (FOP) techniques that solve SHM problems in online real-time structural damage detection for vibrating systems. The following authors performed a novel framework by applying Recursive Principal Component Analysis (RPCA) in conjunction with Time Varying Auto-Regressive Modeling (TVAR) for an online damage detection method for real time processes [13].

A literature review of next-generation smart sensing technology in SHM, such as smartphones, unmanned aerial vehicles (UAVs), cameras, and robotic sensors, are used in acquiring and analyzing the vibration data [14]. A LiDAR (Light Detection and Ranging) device is an instrument that has significant potential for damage detection based on laser scanning providing geometric information about the structures [15].

Although a Light Detection and Ranging (LiDAR) system is highly precise and reliable, the cost of its implementation for SHM tasks can be expensive in the case of a 64-beam model that can cost around $75,000 (USD) [16]. Despite their high cost, LiDAR is mainly used for perception and localization tasks at most high level [17]. The advantage of these systems is that the performance of the system can be determined by using non-destructive techniques (NDT).

This work is focused on a TVS system for displacement measurements. The current TVS has the patent number MX2014000647, which uses a dynamical triangulation method to get angular position and 3D coordinates from objects or surfaces. This system can also perform the same tasks as cameras and LiDAR for SHM tasks. But with advantages like high accuracy, low computational cost, and low volume of data requirement for measurements.

A laser source obtains the geometrical coordinates of a surface under study. A photosensor detects the laser beam reflected. However, in a real operation, interferences of other radiation sources can affect the information collected with a TVS. For example, sunlight is the main interference that should be filtered. For this reason, the reflected laser beam is mixed up with undesired signals.

A novelty signal processing method is proposed for a technical vision system (TVS). A method based on the use of the Shannon entropy for feature extractions of optical patterns in the context of SHM to augment the accuracy of optoelectronic signal classifiers implemented in the metrology subsystem of the TVS. To enhance the TVS spatial coordinate measurement performance at real operation conditions with electrical and optical noisy environments to estimate structural displacement better and evaluate its health.

The following research faces the same problem in reconstructing the real returning signal shape, and its problem is exacerbated by the presence of strong solar background illumination [18]. Using optical filters and higher-power lasers would be a solution. However, these increase the cost of manufacturing a TVS and increase larger usage risks and augment energy consumption. An alternative solution is to apply Artificial intelligence (AI) to detect what signal corresponds to the laser beam. ML can solve the interference issue as a recognition pattern problem. To enhance the accuracy of ML models, Shannon’s entropy is proposed to remove parts that contain random signals and isolate them from the optical patterns.

In this work the following novelty signal processing method is proposed to enhance the TVS accuracy.

A method based on the use of the Shannon entropy for feature extractions of optical patterns in the context of SHM to augment the accuracy of optoelectrical signal classifications implemented in the metrology subsystem of the TVS. To enhance the TVS spatial coordinate measurement performance at real operation conditions with electrical and optical noisy environments to estimate structural displacement better and evaluate its health.

Relevant procedures in the method are:

1. Using a phototransistor with black daylight filter as a photosensor of a TVS to reduce the influence of solar radiation as much as possible.
2. Calibrating the TVS with a turned-off laser and obtaining raw signals (Class 1).
3. Calibrating the TVS with a turned-on laser and obtaining raw signals (Class 2).
4. Creating a Class 3 with data augmentation to create robust ML models.
5. Comparing the performance of five different ML models with LPC and ACC.
6. Comparing the performance of five different ML models with LPC and ACC and Shannon’s entropy as a segmentation process.

This work aims to find the configuration that enhances the performance of ML models to discriminate against sunlight interference. One of the main goals is to implement a pipeline that can recognize the reflected laser beam pattern. For that reason, this research compares the accuracy of five different ML techniques with LPC, ACC, and Shannon’s entropy. The following classifiers such as Naïve Bayes (NB), support vector machines (SVM), linear discriminant analysis (LDA), K-Nearest Neighbors (KNN), and neural network (NN), were used. Data augmentation was implemented to enhance the accuracy of these classifiers.

This paper is organized as follows. Section 2 gives details about the problem statement. Section 3 describes the operational principle of a TVS and the latest improvements. Section 4 provides a brief overview of the feature extractions used. Section 5 summarizes the ML methods used in this work. Section 6 presents the proposed ML pipeline to solve the problem of interference. Section 7 discusses the results and highlights of the experiments carried out in this work. Finally, some conclusions and recommendations from the experiments are shared in Section 8.

## 2. Problem Statement

Recent studies were conducted outdoors and compared with experimentation under indoor (Laboratory) conditions, from which the results showed that undesired signals affected the performance of the TVS. This was primarily due to the conditions of intense radiation [19,20,21]. Consequently, a laser beam cannot be captured by a TVS system. The solar radiation spectrum shows that infrared light is reflected more than ultraviolet (UV) or visible light due to its longer wavelength. This is important to consider because several devices can work with these wavelengths, such as phototransistors (PT) and photodiodes (PD).

PT is more sensitive to light than PD due to high gain. Another advantage of PT over PD is that it can be obtained at low-cost. The PT used in this work minimizes outside interference thanks to its daylight filter. Although PT was chosen, TVS is still detecting low interference outdoors. The interference can be discriminated against using ML models to address this issue. Particular optical patterns only can appear in three different scenarios. The first scenario corresponds to when TVS is turned off. The second scenario appears when TVS is turned on. Finally, the third scenario represents a saturation of a signal captured.

**Figure 1:**
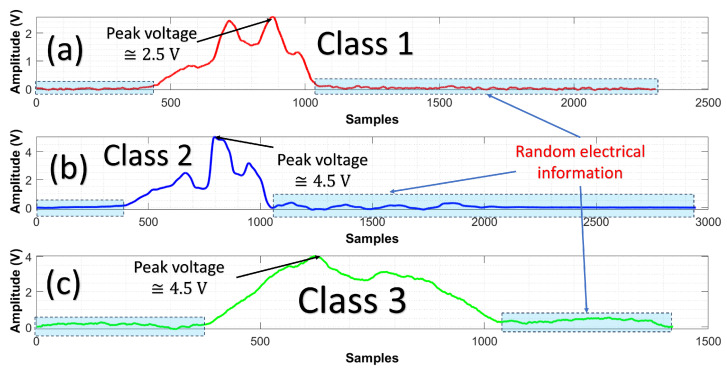
Raw signals mixed up with interference are used for pattern recognition. (**a**) The solid red line represents TVS is turned off. (**b**) Blue color shows the signal captured when TVS is turned on. (**c**) The solid green line signal represents an unknown pattern (this signal was created as a synthetic pattern). Note that an important part of the signal is random electrical information.

Figure 1, shows raw signals detected with the PT. Figure 1a corresponds to the background or possible interferences found outdoors; at that moment, TVS is turned off. This optical pattern has a peak voltage of 2.5 Volts, labeled class 1. The reason is to create an ML model that can discriminate between the interferences and laser scanning of the TVS system. Figure 1b shows a particular pattern at the moment the TVS is turned on. Thanks to laser power, this signal can be captured by the PT. This signal has a peak voltage of 4.5 Volts and is labeled class 2. Note that the peak of voltage of class 1 and class 2 is different. Figure 1c is class 3 created by a data augmentation stage (synthetic signal). This signal represents a random signal created by external factors.

The three classes contain low voltages, captured when radiation is not detected. These are a part of the optical patterns that can be regarded as a problem because there is no relevant information. Random voltage variations are redundant information that needs to be addressed.

## 3. Operational Principle of TVS

In this section, a brief overview of evolution and operational principles of TVS are given.

TVS system is a device that can solve real-time tasks to measure three-dimensional (3D) coordinates. These tasks are needed in many contexts of SHM, such as displacement measurements or surface estimation. This system has two main parts to realize depth measurements. The first component of a TVS is the positioning laser (PL) that uses an active laser in conjunction with mechanical elements, such as a step/servo motor and gears, whereby the space of interest can be radiated. The second component is the scanning aperture (SA), which contains photosensors to receive the radiation reflected from objects under study.

**Figure 2:**
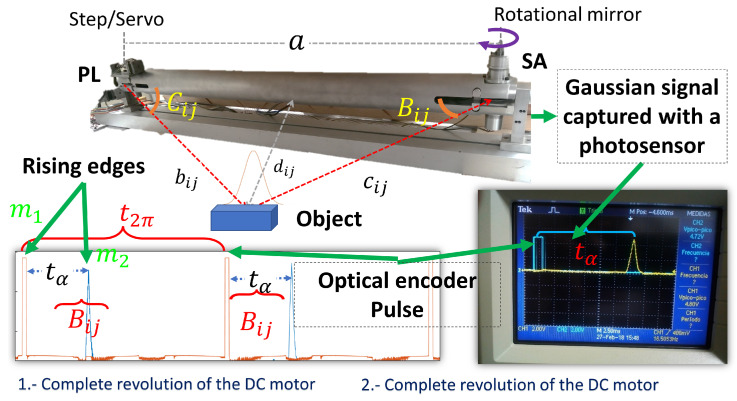
Graphical aid to visualize the main parts of TVS in operation. $B_{i,j}$ is the angular position according to a frame reference. The oscilloscope shows the pulse and a laser beam (Gaussian Signal) without interference.

The distance between PL and SA is known, and can be identified as *a* which is illustrated in Figure 2. Angular position of the PL can be controlled by a step motor or servo motor. PL is an angle known by the user that corresponds to $C_{i,j}$. The angular position of SA is measured by knowing the peak time of the Gaussian signal and period of the DC motor with a speed constant, this is denoted by $B_{i,j}$.

Figure 2 explains how to determine the angular position of SA when a Gaussian signal appears. This signal has the shape of a normal distribution bell (Gaussian). A rotational mirror at an angle of 45° reflects the radiation of an object to the photosensor placed on SA. As a consequence, the Gaussian shape of a signal is formed. The capacitance of a photosensor and signal processing can smooth the Gaussian signal.

Since TVS is scanning at a constant angular velocity, the time elapsed between the start of the first pulse and the second pulse can be used to estimate the angular position. For instance, the angular position β of the PT can be calculated as follows with Equation (1).

(1) $$\begin{array}{c} \beta =2\pi \frac{t_{\alpha }}{T_{2\pi }} \end{array}$$

where the time $t_{\alpha }$ is defined as the interval between the signal $m_{1}$ and the position of the energy center is $m_{2}$.

The local maximum of the Gaussian signal is related to the energetic center of the radiation reflected on the surface studied. A complete revolution of a motor is the period of time used to know the angular position of a local maximum of a Gaussian signal, and this is called $B_{i,j}$. Opto-interrupters or Hall sensors are usually used to calculate the pulses per revolution of a DC motor. In this paper, ITR8102 (Everlight Electronics, New Taipei City, Taiwan) was implemented to know the position of the motor on SA. This opto-interrupter sends pulses for every revolution of the rotational mirror. For each revolution of a DC motor, a Gaussian shape will appear during the scanning, as illustrated in Figure 2.

Knowing the scanning frequency makes it possible to calculate $B_{i,j}$. With this information, the object position is estimated at two different times. If $B_{i,j}$ moves, $d_{i,j}$ can be determined in real-time. Note that distance $d_{i,j}$ corresponds to depth. According to sine theorems and the values of the angles $B_{i,j}$ and $C_{i,j}$ depth information $d_{i,j}$ is estimated with Equation (2).

(2) $$\begin{array}{c} d_{i,j}=a\frac{sin\left(B_{i,j}\right)sin\left(C_{i,j}\right)}{sin[180^{\circ }-\left(B_{i,j}+C_{i,j}\right)]} \end{array}$$

**Figure 3:**
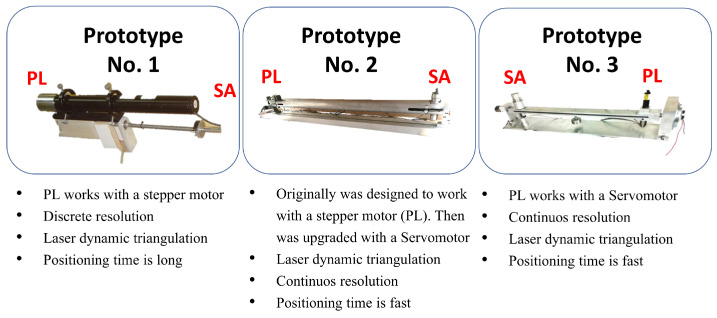
Comparison of different TVS systems developed for measuring 3D coordinates.

Figure 3, details relevant information about different TVS prototypes.

To extend the information of a TVS, the following work [22] shows typical laser scanner constructions and their constraints.

The following researchers [23,24,25] worked with the first version of the TVS prototype number one. They used the TVS for remote sensing and obstacle detection in an unknown environment. This prototype presented simplicity, versatility, and economic accessibility to realize 3D coordinates measurements. An inconvenience of this prototype is that it could only scan in a discontinuous way. In other words, point clouds give shape to the object studied. The next work [26] involved the substitution of the previous (prototype No. 2) and changed the stepper-motor by servo-motors to achieve a continuous laser scan (newly developed prototype No. 3).

A complete mathematical apparatus for processing digital information inside the system and for determining the distances and angle measurements in the system proposed is developed [27].

## 4. Feature Extraction Methods

Feature extractors are mathematical algorithms that recover relevant information (attributes) from a phenomenon like our raw signals captured with a PT. This process is known as feature engineering, and the main purpose is to use representative data with less information. These features enhance the ML models, and redundant data are minimized. There are several methods, such as Autoregressive (AR) Modelling, Linear Predictive Coding (LPC), Autocorrelation coefficients (ACC), Mel Frequency Cepstral Coefficients (MFCC), Fast Fourier transform (FFT), Hilbert transform, just to mention a few. Trends and a zoomed-in perspective of feature extraction methodologies can be consulted in [28].

This work implements ACC and LPC as feature extractors. However, Shannon’s entropy is used to segment only the optical pattern from the electrical signal and remove the rest of the signal.

The description of these techniques are detailed as follows.

### 4.1. Autocorrelation Function

The autocorrelation function (ACF) vector can be used for extract the features (ACC) of the TVS system by measuring the correlation between $y_{t}$ and $y_{t+k}$ where x=0,⋯,k and $y_{t}$ is a stochastic process.

The correlation for lag *k* can be estimated by applying Equation (3). For more details, see the following work [29].

(3) $$\begin{array}{c} \begin{array}{c} r_{k}=\frac{c_{k}}{c_{0}} \end{array} \end{array}$$

where $c_{0}$ represents the sample variance of the time series and $c_{k}$ can be estimated by Equation (4).

(4) $$\begin{array}{c} \begin{array}{c} c_{k}=\frac{1}{T}\sum_{t=1}^{T-k}\left(y_{t}-\bar{y}\right)\left(y_{t+k}-\bar{y}\right) \end{array} \end{array}$$

**Figure 4:**
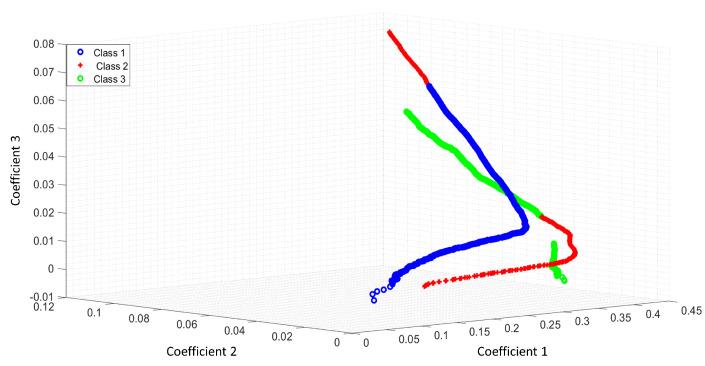
Feature space of ACC as a feature extractor of a raw signal.

Figure 4 shows the feature space of ACC as a feature extractor from a raw signal.

**Figure 5:**
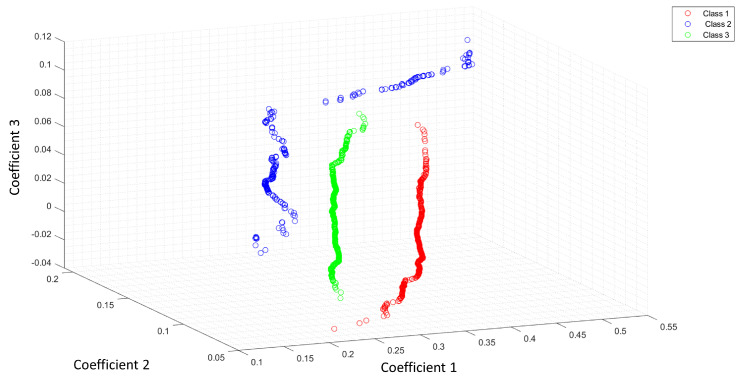
Feature space of ACC and the Shannon entropy as a segmentation process.

Figure 5 shows the feature space of ACC with the Shannon entropy. Note that the segmentation with the Shannon entropy separates each class in comparison with Figure 4. The coefficients 1,2,3 represent the first three features of ACC or LPC. For this study, we extracted 11 features for each optical pattern. These figures differences rely on the useful features extracted with the Shannon entropy as a segmentation process. The ideal case is when the feature extraction process can separate all classes.

### 4.2. Linear Predictor Coefficients

The procedure to calculate the LPC coefficients can be described as follows. First, Equation (5) was applied to calculate the FFT of a desired signal to compute the autocorrelation vector.

(5) $$\begin{array}{c} \begin{array}{c} x_{k}=\sum_{n=1}^{N}x_{n}e^{-j2\pi (k-1)(n-1)/N} \end{array} \end{array}$$

After obtaining FFT $X_{k}$, the inverse discrete Fourier transform of the absolute value of $X_{k}$ value is taken and squared to compute the autocorrelation vector *R* by Equation (6).

(6) $$\begin{array}{c} X_{j}=\frac{1}{n}\sum_{k=1}^{n}Y_{k}e^{2i\pi (j-1)(k-1)/n} \end{array}$$

A scaling is applied to the output and the bias of the autocorrelation is estimated B=R./m, where *m* represents the number of the length of the vector or signal segment under study.

The Hermitian Toeplitz system of equations is built as follows:

(7) $$\begin{array}{c} \left[\begin{array}{cccc} B(1) & B{\left(2\right)}^{*} & \cdots & B{\left(n\right)}^{*}\\ B(2) & B(1) & \cdots & {B\left(n-1\right))}^{*}\\ \vdots & \ddots & \ddots & \vdots \\ B(n) & \cdots & B(2)) & B(1)) \end{array}\right]\left[\begin{array}{c} A(2)\\ A(3)\\ \vdots \\ A(n+1) \end{array}\right]\\ =\left[\begin{array}{c} -B(2)\\ B(3)\\ \vdots \\ -B(n+1) \end{array}\right] \end{array}$$

This system of equations Equation (7) can be solved by Levinson-Durbin, recursion and the real coefficients *A* for the predictor are taken.

**Figure 6:**
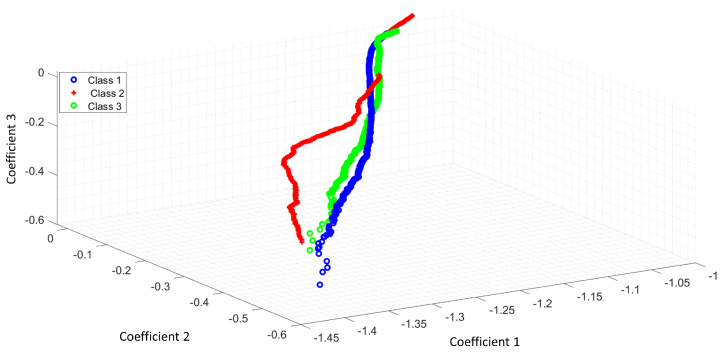
Feature space of LPC as a feature extractor of a raw signal.

**Figure 7:**
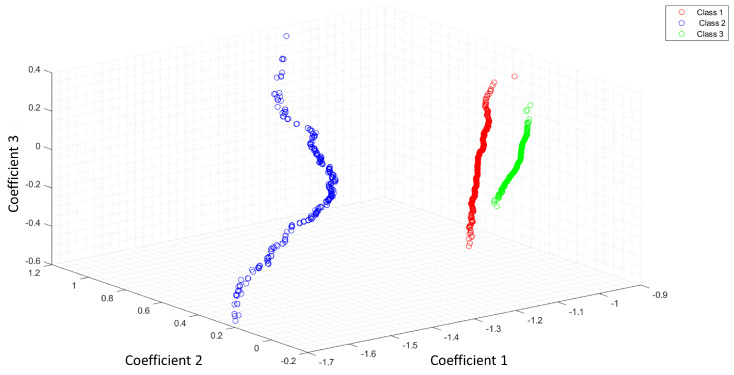
Feature space of LPC and the Shannon entropy as a segmentation process.

Figure 6 illustrates the feature space of LPC as a feature extractor from a raw signal. Figure 7 shows how the classes were separated with Shannon’s entropy segmentation process.

### 4.3. Entropy as an Optical Feature

Entropy is a broad concept that measures the disorder in a random system. According to Shannon, a non-linear measurement in dynamic signals, measures the average information contents associated with the data randomness encountered in a signal or event [30]. The relevance of SHM is that SE is a useful tool to show changes in the measured signals associated with the structure condition.

Given a source of random events from the discrete set of possible events $a_{1},a_{2},\cdots ,a_{n},$ with associated probability distribution $P\left(a_{1}\right),P\left(a_{2}\right),\cdots ,P\left(a_{n}\right)$, the average information per source output can be called as the entropy of the source, see Equation (8)

(8) $$\begin{array}{c} H=-\sum_{n=1}^{N}P\left(a_{n}\right)logP\left(a_{n}\right) \end{array}$$

*H* may alternatively be understood as a measure of unpredictability of information content [31].

In the case of this work, Shannon’s entropy is used as a segmentation process. As mentioned before, part of the optical patterns are random electrical signals that increase the size of the dataset. It measures the entropy of a signal divided into frames with 80 samples of window length and with 30 samples of overlap.

## 5. Machine Learning Classifiers

The power of ML techniques is based on the algorithm chosen to solve a complex problem. The following ML classifiers, such as NB, SVM, LDA, KNN, and NN, were selected to discriminate between the reflected laser beam and sunlight or other radiation sources. These techniques are described below.

### 5.1. Naives Bayes

NB is a probabilistic classifier that works well as most distributions of related features follow probabilistic nature [32]. This classifier assumes that the properties of the features on a given class are independent of the values of the other features. By knowing our class labels and training data set as $T=\left(x_{1},y_{1}\right),\cdots \left(x_{N},y_{N}\right)$. Each instance from the data set is represented by *n*-dimensional feature vector, $X=x_{1},x_{2},\ldots ,x_{n}$. Each label is represented by y∈{1,⋯,K}, where *K* is the number of a class. In this work there are three classes, $C_{1},C_{2}$ and $C_{3}$.

According to [33], given a sample *X*, NB will predict that *X* belongs to the class having the highest a posteriori probability, which is conditioned on *X*. *X* is predicted to belong to the class $C_{i}$ if and only if.

(9) $$\begin{array}{c} P(C_{i}|X)>P(C_{j}|X)\;for\;1\leq j\leq m,\;j\neq i \end{array}$$

Based on Bayes’ theorem,

(10) $$\begin{array}{c} P(C_{i}|X)=\frac{P(X|C_{i})P\left(C_{i}\right)}{P(X)} \end{array}$$

where $P(C_{i}|X)$ is the class prior probability and posteriori probability. $P(X|C_{i})$ is the likelihood, which is the probability of the predictor given class.

### 5.2. Support Vector Machines

A SVM classifier starts with a construction of a decision function f(x,ω)=sign(h(x,ω)) with outputs {±1}, where h(x,ω)) is a separating hyperplane that can be expressed as follows:

(11) $$\begin{array}{c} h(x,\omega )=\langle \omega +\phi (x)\rangle +b \end{array}$$

where ω defines a direction perpendicular to the hyperplane.

Although SVM was initially designed for binary classification, several methods have been proposed to create a multiclass classifier. One-versus-rest (1VR) and one-versus-one (1V1) are representative ensemble schemes for discrimination for more than two categories. This approach’s main issue is constructing a good Error Correcting Output Codes (ECOC) matrix [34]. This work is based on 1V1 that fits K(K−1)/2 by individual binary classifiers SVM models.

One of the main problems of applying multi-class classification is usually solved by a decomposing and reconstruction procedure when two-class decision machines are implied [35].

### 5.3. Linear Discriminant Analysis

An extension of Fisher’s linear discriminant for *n* classes can be represented as an intra-class matrix according to [36].

(12) $$\begin{array}{c} \begin{array}{c} {\hat{\Sigma }}_{w}=S_{1}+\cdots +S_{n}=\sum_{i=1}^{n}\sum_{x\in c_{i}}\left(x-\bar{x_{i}}\right){\left(x-\bar{x_{i}}\right)}^{{}^{\prime }} \end{array} \end{array}$$

where $\bar{x}$ is the mean value and $\bar{x_{i}}$ corresponds to the mean for each class.

The inter-class matrix can be represented as follows:

(13) $$\begin{array}{c} \begin{array}{c} {\hat{\Sigma }}_{b}=\sum_{i=1}^{n}m_{i}\left(\bar{x_{i}}-\bar{x}\right){\left(\bar{x_{i}}-\bar{x}\right)}^{{}^{\prime }} \end{array} \end{array}$$

where $m_{i}$ represents the number of training samples for each class.

### 5.4. K-Nearest Neighbors

KNN is one of the most popular algorithms to be used as a multi-class ML technique. This technique is a non-probabilistic classifier as well as SVM. It is well known as lazy learning because it does not carry out a training phase [37]. The latest trends and applications of KNN in Big Data [38] are used in the context of smart cities [39]. This algorithm compares the *k* nearest neighbors to be used as a decision rule to classify a new distance as belonging to a class. Furthermore, KNN can be applied for regression problems. KNN algorithm is a distance-based classifier, and the main functions employed by this algorithm are Euclidean, Mahalanobis, Hamming, and Citiblock. The Euclidean distance is used in this work. This metric can be expressed as follows.

(14) $$\begin{array}{c} \begin{array}{c} d\left(x,y\right)=\sum_{i=1}^{N}\sqrt{x_{i}^{2}-y_{i}^{2}} \end{array} \end{array}$$

where, *N* is the total number of samples.

### 5.5. Neural Network

Learning and memory are complex processes that AI tries to imitate or understand. An artificial NN is a computational tool inspired by human brain behavior to perform these tasks. Recent development in neural networks profoundly showed incredible results in object classification, pattern recognition, and natural language processing [40].

A NN can be classified into two categories such as feed-forward (FNN) and feed-backward or recurrent (RNN), according to their interconnection between the neuron layers.

Convolutional neural networks (CNN) are another type of NN well-known by machine vision designers. The NN classifier used in this work is based on FNN.

Weight matrix and activation functions are important parts of designing a NN. The activation function transforms an input signal into an output signal. The functions such as Rectified Linear Unit (RELU), SoftMax, Binary Step Function, Linear, Sigmoid, Tanh, Exponential Linear Unit, and Swish are commonly used.

A RELU function has the main goal of establishing a threshold operation for input, and this can be expressed as follows:

(15) $$\begin{array}{c} \begin{array}{c} f\left(x\right)=\left\{\begin{array}{c} 0\;if\;x<0\\ x\;if\;x\geq 0 \end{array}\right. \end{array} \end{array}$$

The SoftMax activation function regularly is applied to the final fully connected layer. This function can be implemented as follows:

(16) $$\begin{array}{c} \begin{array}{c} softmax\left(z_{i}\right)=\frac{exp(z_{i})}{\sum_{j=1}^{K}exp\left(z_{i}\right)} \end{array} \end{array}$$

where *z* are the real values of the output layer and *K* is the number of classes. SoftMax functions convert these values into probabilities (classification scores).

The parameters of activation functions applied in this work are to create a NN classifier using two fully connected layers, each with three outputs. The RELU activation function was implemented for every fully connected layer of the NN model. As an output layer of the NN classifier, a SoftMax activation function was used.

The following authors [41], implemented an FNN application to evaluate sustainable urban environmental quality to deal with air pollution. Ref. [42] reviews on the basic theories and recent algorithms for optimizing NN.

## 6. Proposed Classification Schemes

**Figure 8:**
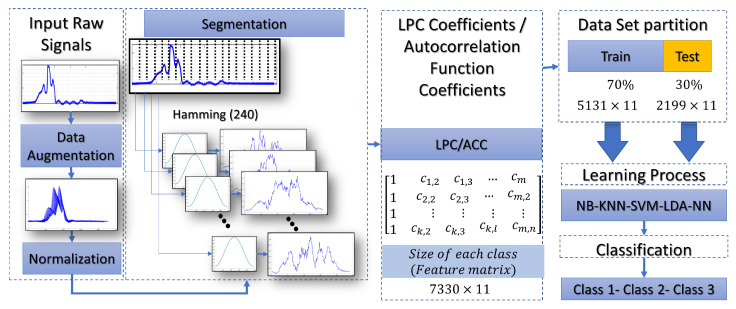
Flow chart of the reflected laser beam classification without Shannon’s entropy for removing frames.

**Figure 9:**
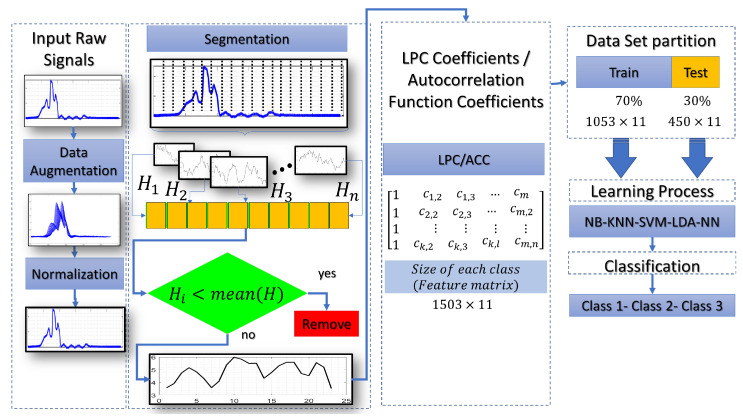
Flow chart of the laser beam classification based on Shannon’s entropy segmentation.

This section gives an overview of a practical implementation of ML with different techniques. Figure 8, shows the complete procedure for classifying the laser beam. This procedure starts with inputting of raw signal captured with a phototransistor (peak wavelength 940 nm). A data augmentation process was used to enhance the performance of the ML models. In that stage, time scale modification (TSM) is applied on the input raw signals by applying different speedup factors. In the literature, this is known as alpha. Then, a white Gaussian noise is added to a raw signal. Finally, the signal is filtered a mean filter by using 14 coefficients. Different signals were created with this procedure to make more robust predictions. The normalization process is realized to work with uniform data with values between 0 and 1. The main goal of the segmentation process is to divide the input signal into several windows, and each segment should also be meaningful. Particular features of each window are evident thanks to this process. As shown in Figure 8, a Hamming window of a 240-point is created to convolve with each segment. The following process shows that ACC or LPC are estimated and stored in a feature matrix. This matrix is split into two data sets. Training and test data sets are used in the learning process. Finally, the classification process gives the accuracy of the feature vectors tested. Figure 9, shows the procedure of how the optical patterns were collected. Each frame has an entropy value representing the optical pattern’s randomness level. Smaller values of total entropy are removed. Note that in Figure 9 the size of the feature matrix with Shannon’s entropy is reduced in comparison with Figure 8. Appendix A shows the pseudocode of two proposed classification schemes, as described previously.

## 7. Results and Discussion

This section explains how the results of this study were obtained to classify the optical patterns into class 1, class 2 and class 3 (see Figure 1 as reference for each kind of class).

### 7.1. Results without Entropy Segmentation

Table 1 shows the percentages of correctly and incorrectly classified instances for each true class carried out in this experiment. True positives of class 1, class 2, and class 3 were 1386, 1970 and 1495, respectively. The confusion matrix shows that 1746 measurements were misclassified. The class precision reached for class 1, class 2, and class 3 was 63%, 89.6%, and 68%, respectively. The accuracy of correct classifications overall test optoelectrical signals was 73.53% while the test error was 26.47%. In the case of NB-ACC the accuracy of correct classifications overall test optoelectrical signals was 57.48% while the test error was 42.52%. The confusion matrix shows that 2805 measurements were misclassified.

Table 2 indicates the percentages of correctly and incorrectly classified instances for each true class in this experiment. True positives of class 1, class 2, and class 3 were 2199, 2199, and 2172, respectively. All the measurements belonging to classes 1 and 2 are classified correctly. The confusion matrix shows that 27 measurements were misclassified. The classification accuracy reached for class 1, class 2, and class 3 was 100%, 100%, and 98.8%, respectively. The accuracy of correct classifications overall test optoelectrical signals was 99.59% while the test error was 0.41%. In the case of NB-ACC, the accuracy of correct classifications overall test optoelectrical signals was 74.40% while the test error was 25.60%. The confusion matrix shows that 1689 measurements were misclassified.

Table 3 shares the percentages of correctly and incorrectly classified instances for each true class and predicted class in this experiment. True positives of class 1, class 2, and class 3 were 2198, 2199, and 2181, respectively. All the measurements belonging to class 2 are classified correctly. The confusion matrix shows that 19 measurements were misclassified. The class precision reached for class 1, class 2, and class 3 was 99.99%, 100%, and 99.2%, respectively. The accuracy of correct classifications overall test optoelectrical signals was 99.71% while the test error was 0.29%. In the case of NB-ACC, the accuracy of correct classifications overall test optoelectrical signals was 99.51% while the test error was 0.49%. The confusion matrix shows that 32 measurements were misclassified.

Table 4 indicates the percentages of correctly and incorrectly classified instances for each true class and predicted class carried out in this experiment. The configurations KNN-LPC and KNN-ACC achieved the same results. True positives of class 1, class 2, and class 3 were 2199, 2199, and 2199, respectively. The classification accuracy reached for class 1, class 2, and class 3 was 100%, 100%, and 100%, respectively. All the measurements belonging to each class were classified correctly. The accuracy of correct classifications overall test optoelectrical signals was 100% while the test error was 0%.

Table 5 shows the percentages of correctly and incorrectly classified instances for each true class and predicted class carried out in this experiment. The configuration NN-LPC achieved the following results. True positives of class 1, class 2, and class 3 were 2199, 0, and 0, respectively. The classification accuracy reached for class 1, class 2, and class 3 was 100%, 0%, and 0%, respectively. All the measurements belonging to classes 2 and 3 were classified incorrectly. The confusion matrix shows that 4398 measurements were misclassified. The accuracy of correct classifications overall test optoelectrical signals was 33.33% while the test error was 66.67%.

In the case of NN-ACC the accuracy of correct classifications overall test optoelectrical signals was 100% while the test error was 0%. All the measurements belonging to each class were classified correctly.

### 7.2. Results Entropy Segmentation

Table 6 shows the percentages of correctly and incorrectly classified instances with NB-SH-LPC model. The accuracy of correct classifications overall test optoelectrical signals was 95.33%, while the test error was 4.67%. The confusion matrix shows that 21 measurements were misclassified.

In the case of SHH-NB-ACC, the accuracy of correct classifications overall test optoelectrical signals was 97.56% while the test error was 2.44%. The confusion matrix shows that 11 measurements were misclassified.

Table 7 shows the percentages of correctly and incorrectly classified instances with the SVM-SH-LPC model. All the measurements belonging to each class were classified correctly. The accuracy of correct classifications overall test optoelectrical signals was 100% while the test error was 0%.

In the case of SVM-SH-ACC, the accuracy of correct classifications overall test optoelectrical signals was 99.56% while the test error was 0.44%. The confusion matrix shows that two measurements were misclassified.

Table 8 and Table 9 show the percentages of correctly and incorrectly classified instances with LDA-SH-LPC, LDA-SH-ACC models, KNN-SH-LPC, KNN-SH-ACC models. All the measurements belonging to each class were classified correctly. The accuracy of correct classifications overall test optoelectrical signals was 100% while the test error was 0%.

Table 10 shows the percentages of correctly and incorrectly classified instances with the NN-SH-LPC model. All the measurements belonging to each class were classified correctly. The accuracy of correct classifications overall test optoelectrical signals was 100% while the test error was 0%. In the case of NN-SH-ACC, the accuracy of correct classifications overall test optoelectrical signals was 99.33% while the test error was 0.67%. The confusion matrix shows that three measurements were misclassified.

**Figure 10:**
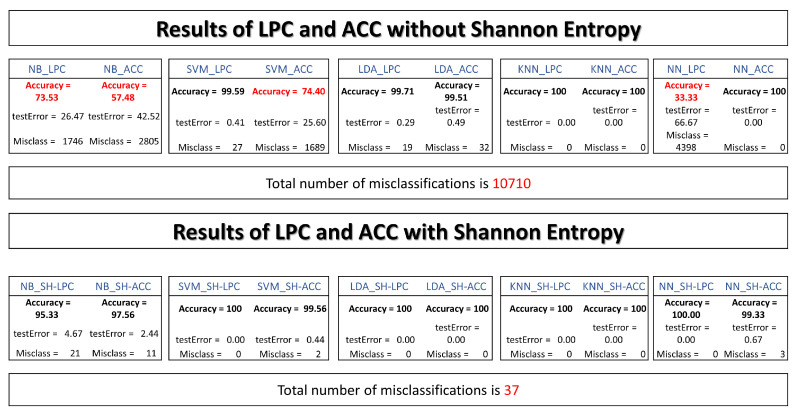
A summary of machine learning model’s performance. The first results do not include the Shannon entropy (SH) as a segmentation process. The second results show the performance of ML models with the SH segmentation method.

Figure 10, summarizes the performance of the ML models with ACC, LPC, and Shannon’s entropy. Without Shannon’s entropy segmentation, the overall misclassification reached was 10710. This pipeline needed better results in terms of accuracy for NB-LPC, NB-ACC, SVM-ACC, and NN-LPC. The worst accuracy was 33.33%.

With Shannon’s entropy as a segmentation process, the overall misclassification was 37. The accuracy of the ML classifiers was superior to the previous pipeline.

## 8. Conclusions

Optical sensors can be viewed as an effective transducer to collect data without physically contacting the object under study. Significant value added for remote sensing, such as quantitative or qualitative information, can be gathered. Accessing this information enables us to study the optimal UI parameters, measure many variables, and design early warning systems. Using a phototransistor as a transducer leads to reasonable results in detecting laser beams. The advantage of using this sensor is the internal daylight-blocking filter, which can be bought at low-cost. The main contribution of this study is related to the electronic and physical aspects of sensors in urban sensing systems for SHM tasks and the application of ML methods for its enhancement.

In this paper, we have shown how a TVS can be enhanced using an IT approach. The proposed approach of using an ML framework was implemented to solve the problem related to interference in a real environment. Feature extraction methods were used as a preprocessing stage, and various classifiers were reviewed. The windowing process was integrated into the ML pipeline to split the input signal into temporal segments. The Shannon entropy was used to remove and extract meaningful information from optical patterns.

Results showed significantly better accuracy using Shannon’s entropy as a segmentation process. Relevant information was extracted from signals, and ML models were created. Accuracy reached using SVM, LDA, KNN, and NN was over 99%. The accuracy of NB-SH was 95.33% and 97.56% with LPC and ACC, respectively. These results demonstrate Shannon entropy superiority in extracting the optical patterns over frames of complete segments. Without Shannon’s entropy segmentation, the worst accuracy was 33.33%.

Practical implementation of this frame can avoid outdoor interferences. In addition, these findings provide additional information about the type of ML techniques that can be used in outdoors environments. This work can be extended to other applications. Three different ECG signals were classified to validate these configurations with Shannon’s entropy and LPC-ACC, showing satisfactory results. This indicates that the ML framework with LPC-ACC and the Shannon entropy can solve pattern recognition problems.

## Figures and Tables

**Table 1:** Performance of NB-LPC and NB-ACC.

Predicted Class NB-LPC					Predicted Class NB-ACC				
		**Class1**	**Class2**	**Class3**			**Class1**	**Class2**	**Class3**
	Class1	1386	0	813		Class1	1253	894	52
True	Class2	0	1970	229	True	Class2	17	2182	0
	Class3	704	0	1495		Class3	730	1112	357

**Table 2:** Performance of SVM-LPC and SVM-ACC.

Predicted Class SVM-LPC					Predicted Class SVM-ACC				
		**Class1**	**Class2**	**Class3**			**Class1**	**Class2**	**Class3**
	Class1	2199	0	0		Class1	1212	987	0
True	Class2	0	2199	0	True	Class2	23	2176	0
	Class3	27	0	2172		Class3	228	451	1520

**Table 3:** Performance of LDA-LPC and LDA-ACC.

Predicted Class LDA-LPC					Predicted Class LDA-ACC				
		**Class1**	**Class2**	**Class3**			**Class1**	**Class2**	**Class3**
	Class1	2198	1	0		Class1	2197	2	0
True	Class2	0	2199	0	True	Class2	9	2190	0
	Class3	18	0	2181		Class3	1	20	2178

**Table 4:** Performance of KNN-LPC and KNN-ACC.

Predicted Class KNN-LPC					Predicted Class KNN-ACC				
		**Class1**	**Class2**	**Class3**			**Class1**	**Class2**	**Class3**
	Class1	2199	0	0		Class1	2199	0	0
True	Class2	0	2199	0	True	Class2	0	2199	0
	Class3	0	0	2199		Class3	0	0	2199

**Table 5:** Performance of NN-LPC and NN-ACC.

Predicted Class NN-LPC					Predicted Class NN-ACC				
		**Class1**	**Class2**	**Class3**			**Class1**	**Class2**	**Class3**
	Class1	2199	0	0		Class1	2199	0	0
True	Class2	2199	0	0	True	Class2	0	2199	0
	Class3	2199	0	0		Class3	0	0	2199

**Table 6:** Performance of NB-SH-LPC and NB-SH-ACC.

Predicted Class NB-SH-LPC					Predicted Class NB-SH-ACC				
		**Class1**	**Class2**	**Class3**			**Class1**	**Class2**	**Class3**
	Class1	143	7	0		Class1	144	6	0
True	Class2	0	150	0	True	Class2	0	150	0
	Class3	12	2	136		Class3	3	2	145

**Table 7:** Performance of SVM-SH-LPC and SVM-SH-ACC.

Predicted Class SVM-SH-LPC					Predicted Class SVM-SH-ACC				
		**Class1**	**Class2**	**Class3**			**Class1**	**Class2**	**Class3**
	Class1	150	0	0		Class1	148	0	2
True	Class2	0	150	0	True	Class2	0	150	0
	Class3	0	0	150		Class3	0	0	150

**Table 8:** Performance of LDA-SH-LPC and LDA-SH-ACC.

Predicted Class LDA-SH-LPC					Predicted Class LDA-SH-ACC				
		**Class1**	**Class2**	**Class3**			**Class1**	**Class2**	**Class3**
	Class1	150	0	0		Class1	150	0	0
True	Class2	0	150	0	True	Class2	0	150	0
	Class3	0	0	150		Class3	0	0	150

**Table 9:** Performance of KNN-SH-LPC and KNN-SH-ACC.

Predicted Class KNN-SH-LPC					Predicted Class KNN-SH-ACC				
		**Class1**	**Class2**	**Class3**			**Class1**	**Class2**	**Class3**
	Class1	150	0	0		Class1	150	0	0
True	Class2	0	150	0	True	Class2	0	150	0
	Class3	0	0	150		Class3	0	0	150

**Table 10:** Performance of NN-SH-LPC and NN-SH-ACC.

Predicted Class NN-SH-LPC					Predicted Class NN-SH-ACC				
		**Class1**	**Class2**	**Class3**			**Class1**	**Class2**	**Class3**
	Class1	150	0	0		Class1	147	1	2
True	Class2	0	150	0	True	Class2	0	150	0
	Class3	0	0	150		Class3	0	0	150

**Figure A1:**
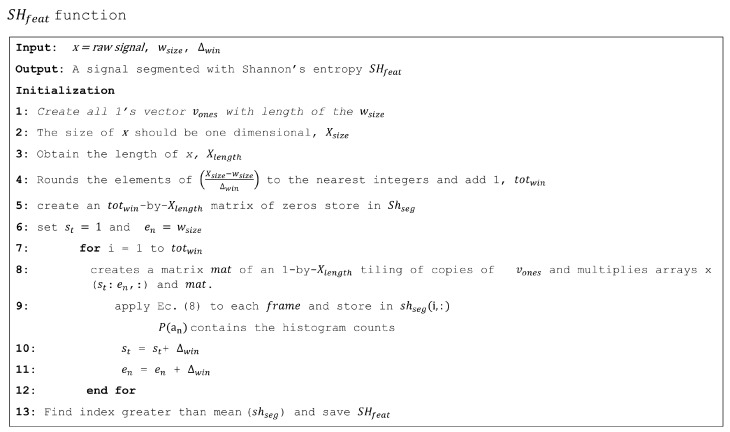
Example of pseudocode to extract features with the Shannon Entropy method. This code is for one-dimensional digital signal processing.

**Figure A2:**
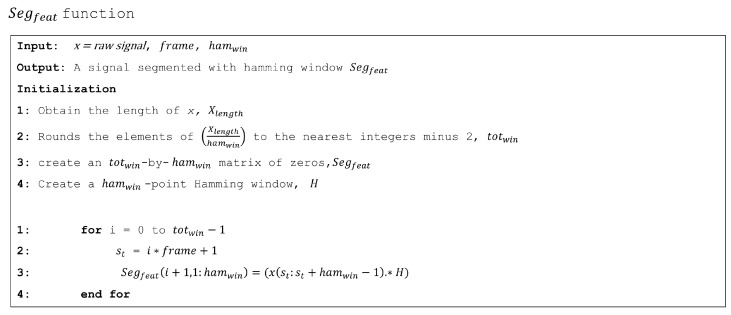
Example of pseudocode to extract features with a Hamming Window applied to each frame. This is for one-dimensional signals. This code is for one-dimensional digital signal processing.

**Figure A3:**
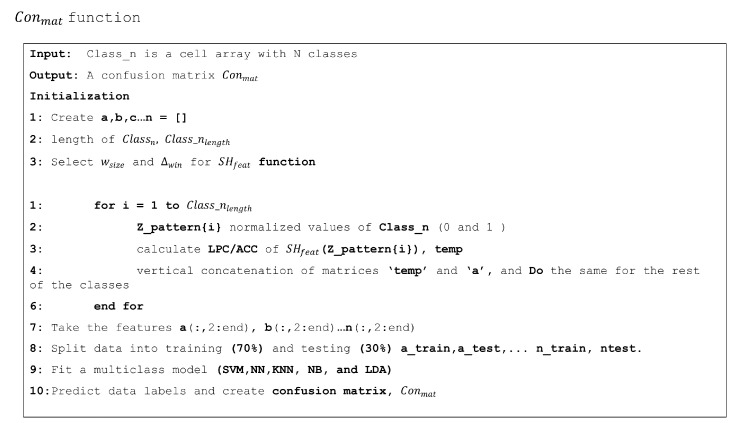
This code shows the procedures to build the ML models. ML designers should select LPC or ACC to conduct the experiments. Note that this pseudo code needs the function of $SH_{feat}$.

**Figure A4:**
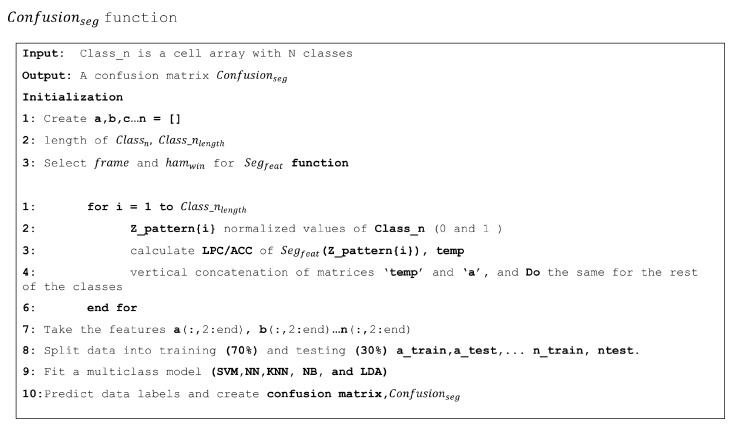
This code shows the procedures to build the ML models. ML designers should select LPC or ACC to conduct the experiments. Note that this pseudo code needs the function of $Seg_{feat}$.

## Data Availability

Not applicable.

## Appendix A. Pseudocode for the Important Parts of the Experimental Design

Figure A1, Figure A2, Figure A3 and Figure A4 show a general framework for classifying optical patterns. These procedures were adopted to conduct the experiments.
